# Optimizing Colorimetric Assay Based on V_2_O_5_ Nanozymes for Sensitive Detection of H_2_O_2_ and Glucose

**DOI:** 10.3390/s16040584

**Published:** 2016-04-22

**Authors:** Jiaheng Sun, Chunyan Li, Yanfei Qi, Shuanli Guo, Xue Liang

**Affiliations:** School of Public Health, Jilin University, Changchun 130021, China; sunjh15@mails.jlu.edu.cn (J.S.); licy14@mails.jlu.edu.cn (C.L.); guosl15@mails.jlu.edu.cn (S.G.); liangxue2714@mails.jlu.edu.cn (X.L.)

**Keywords:** V_2_O_5_ nanozymes, variation, substrates, H_2_O_2_, glucose

## Abstract

Nanozyme-based chemical sensing is a rapidly emerging field of research. Herein, a simple colorimetric assay for the detection of hydrogen peroxide and glucose based on the peroxidase-like activity of V_2_O_5_ nanozymes has been established. In this assay, the effects of pH, substrate, nanozyme concentrations and buffer solution have been investigated. It was found that compared with 3,3′,5,5′-tetramethylbenzidine (TMB), the enzyme substrate *o*-phenylenediamine (OPD) seriously interfered with the H_2_O_2_ detection. Under the optimal reaction conditions, the resulting sensor displayed a good response to H_2_O_2_ with a linear range of 1 to 500 μM, and a detection limit of 1 μM at a signal-to-noise ratio of 3. A linear correlation was established between absorbance intensity and concentration of glucose from 10 to 2000 μM, with a detection limit of 10 μM. The current work presents a simple, cheap, more convenient, sensitive, and easy handling colorimetric assay.

## 1. Introduction

Nanozymes, as the next-generation artificial enzymes, have attracted wide interest in recent years. Compared with natural enzymes, nanozymes, with their advantages of high stability against denaturing, low-cost, easy storage and treatment are attractive and promising candidates in chemical sensing, immunoassay development, cancer diagnostics and therapy, and environmental protection [1]. At present, a large number of nanoparticle (NP) artificial enzymes have been constructed to mimic natural enzymes, including iron oxide-based NPs with peroxidase and catalase-like activities [2,3], cerium oxide-based nanomaterials with oxidase, catalase and SOD mimetic properties [4,5], cobalt oxide ones that are peroxide and catalase mimics [6,7], copper oxide and manganese dioxide nanomaterials that display oxidase-like activity [8,9], vanadium pentoxide peroxidase mimics [10], and metal/bimetal-based [11] and carbon-based NPs [12] with oxidase, peroxidase, and SOD mimetic activity. Thereinto, vanadium pentoxide nanowires had aroused special attention due to their activity towards peroxidase substrates and long-term antibiofouling capabilities [13,14]. However, to date, no facile, fast and ultrasensitive detection methods have been developed based on the peroxidase catalytic activity of vanadium pentoxide nanowires. Additionally, more research is needed on the nanozyme catalytic reaction for biological and chemical analysis.

Hydrogen peroxide (H_2_O_2_), an essential oxidizing agent, plays an important role in the food production, biomedicine, pharmaceutical, industrial and environmental fields [15]. It was proved to be a byproduct of metabolic oxidation processes, and it is immediately dangerous to life and health when its concentration reaches 75 ppm [16]. Glucose is one of the essential nutrients and an important source of energy for human life via its *in vivo* metabolism. Blood glucose levels in healthy humans stay within narrow limits throughout the day (4.0–8.0 mmol/L) [17]. Glucose is also widely used in the food and pharmaceutical industry, therefore, quantitative determination of glucose is significant for industrial quality control and processing applications [18]. Most glucose biosensors are based on the oxidation or reduction of enzymatically produced H_2_O_2_ [19,20,21,22]. Hence, the detection of H_2_O_2_ concentrations has attracted the interest of many chemists for a long time. Until now, several techniques have been developed for a reliable and sensitive determination of H_2_O_2_, such as chemiluminescence [23], fluorometry [24], liquid chromatography [25] and electrochemistry [26]. Among these methods, sensitive spectrometric methods for the H_2_O_2_ determination based on the horseradish peroxidase (HRP)-catalyzed enzymatic reactions have attracted substantial attention owing to the rapid response, and low fabrication cost characteristics [27,28]. Although HRP has high specificity and sensitivity, the application of the free natural enzyme is limited by its poor stability, and high cost [29].

In this paper, a simple colorimetric assay for the detection of hydrogen peroxide and glucose based on the peroxidase-like activity of V_2_O_5_ nanozymes has been established. In this assay, the effects of pH, substrate, nanozymes concentrations and buffer solution have been investigated. Under the optimal reaction conditions, the resulting biosensor displayed a good response to H_2_O_2_ and glucose. In addition, it exhibited excellent anti-interference ability and fast response. The colorimetric assay based on V_2_O_5_ nanozymes may be broadly applicable in clinical diagnoses and monitoring environmental water pollution.

## 2. Materials and Methods

### 2.1. Chemicals and Materials

All the chemicals were of analysis grade and used without further purification. 3,3′,5,5′-Tetramethylbenzidine (TMB) was purchased from Tokyo Chemical Industry Co., Ltd. (Tokyo, Japan). Glucose oxidase (GOx) was purchased from Aladdin Reagent Co., Ltd. (Shanghai, China). *o*-Phenylenediamine (OPD) was purchased from Tianjin Guangfu Fine Chemical Research Institute (Tianji, China). VOSO_4_, KBrO_3_, glucose and 30% H_2_O_2_, *etc.* were purchased from Beijing Chemical Works (Beijing, China).

### 2.2. Synthesis of V_2_O_5_ Nanozymes

V_2_O_5_ nanozymes were synthesized according to a literature procedure [30], with minor adjustments. In brief, VOSO_4_·nH_2_O (2.4 mmol) and KBrO_3_ (1.5 mmol) were dissolved in distilled water (9 mL) and stirred for 30 min at room temperature. Then the solution was transferred to a Teflon-lined stainless steel autoclave, which was maintained at 180 °C for 24 h. After the sample was cooled to room temperature naturally, the resulting dark yellow precipitates were filtered off, washed with distilled water and ethanol several times, and dried in an oven at 80 °C.

### 2.3. Physical Characterization

The V_2_O_5_ nanozymes were thoroughly characterized by various methods. Transmission electron microscopy (TEM) observation was performed using a 100CX transmission electron microscope (JEOL, Tokyo, Japan) with an acceleration voltage of 100 kV. The Fourier Transform Infrared (FTIR) spectra were recorded in the range 400–4000 cm^−1^ on an Alpha Centauri FT/IR spectrophotometer (Nicolet, Denver, CO, USA) using KBr pellets. The X-ray powder diffraction method was carried out in a D/max-rA power diffractometer (Rigaku, Tokyo, Japan) using Cu-Kα monochromatic radiation (λ = 1.5418 Å).

### 2.4. H_2_O_2_ Detection Using V_2_O_5_ Nanozymes

With OPD or TMB as substrate, the H_2_O_2_ sensing assay was performed as follows: V_2_O_5_ nanozymes solution (1 mM, 60 μL) was added into NaOAc-HOAc buffer solution (pH = 4.0, 2400 μL), followed by the addition of TMB (or OPD) solution (1.5 mM in ethanol, 480 μL) and H_2_O_2_ (30%, 60 μL). The UV/Vis spectra were recorded at 660 nm for TMB and 450 nm for OPD after reaction for 5 min *vs.* a blank containing only the substrate solution. The buffer solutions from pH 3.0 to 8.0 and different reaction buffers were investigated, under conditions identical to those used above. All assays were done at 25 °C unless otherwise indicated, and analysed with a UV-Vis spectrophotometer (Metash Instruments Inc., Shanghai, China).

### 2.5. Glucose Detection Using V_2_O_5_ Nanozymes

Glucose detection was performed as follows: GOx (1 mg/mL, 200 μL), glucose of different concentrations (200 μL), and PBS (pH = 7.0, 400 μL) were incubated at 37 °C for 60 min. TMB (1.5 mM in ethanol, 400 μL), V_2_O_5_ nanozymes solution (50 μL), and NaOAc-HOAc buffer solution (pH = 4.0, 1750 μL) were added to the above glucose reaction solution. Then the concentrations of glucose were measured after the mixed solution incubated for 5 min *vs.* a blank containing only the substrate solution. For glucose detection in blood, the blood sample was firstly centrifuged at 12,000 rpm for 5 min. Then, the supernatant (200 μL) was diluted to 400 μL using PBS (pH = 7.0) before subsequent use. These diluted supernatants were then used with GOx for glucose detection as described above instead of glucose aqueous solution.

## 3. Results and Discussion

### 3.1. Characterization of V_2_O_5_ Nanozymes

**Figure 1:**
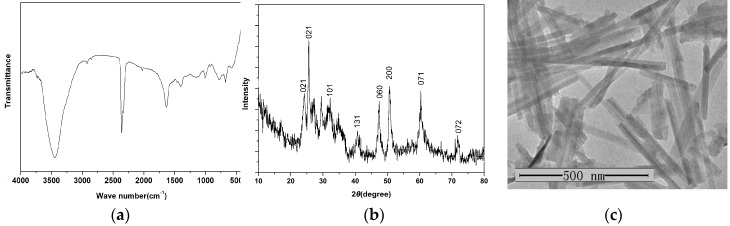
Characterizations of V_2_O_5_ nanozymes: (**a**) FTIR spectrum; (**b**) XRD pattern; (**c**) TEM images.

The physical characterization data of the V_2_O_5_ nanozymes is shown in Figure 1. The FTIR spectrum can be seen in Figure 1a. The bands at about 1000 cm^−1^ come from bending vibrations of the V-O bridge bonds. The formation of orthorhombic V_2_O_5_ nanozymes is confirmed from the X-ray diffraction pattern (Figure 1b). Transmission electron microscopy (TEM) images indicate nanozymes of different sizes with lengths of ~500 nm (Figure 1c).

### 3.2. Principle

In pH 4 NaOAc-HOAc buffer solutions at 25 °C, V_2_O_5_ nanozymes of 500 nm mean size catalyzed the oxidation of 3,3′,5,5′-tetramethylbenzidine (TMB) and *o*-phenylenediamine (OPD) enzyme substrates in the presence of H_2_O_2_ to produce blue and orange colors, respectively. With increased H_2_O_2_ increased concentration more TMB and OPD will be oxidized by more H_2_O_2_, so the absorbance intensity becomes stronger. The absorbance intensity shows a linear dependence on the concentration of H_2_O_2_ and related materials.

**Figure 2:**
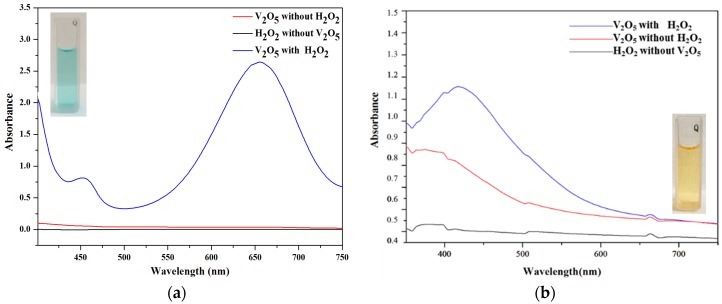
The catalytic effect of V_2_O_5_ nanozymes with TMB (**a**) and OPD (**b**) as the substrate in the presence of H_2_O_2_.

The UV/vis spectra are shown in Figure 2. It can be seen that substrate solutions in the presence of H_2_O_2_ or V_2_O_5_ exhibit no strong absorption, however, strong absorption peaks were found at 452 nm, 660 nm for TMB (Figure 2a), and 450 nm for OPD (Figure 2b) when the H_2_O_2_ and V_2_O_5_ were added into the system.

### 3.3. Effect of pH

**Figure 3:**
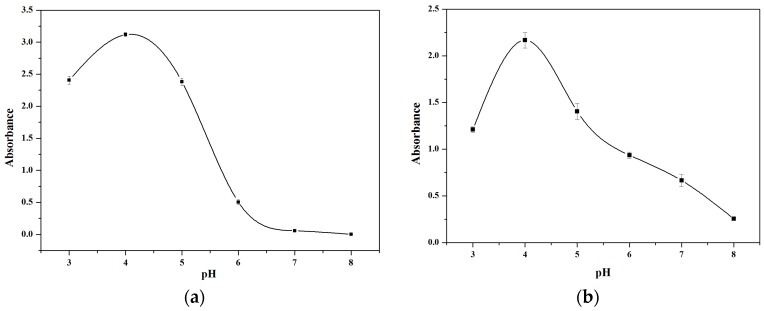
Effects of pH with TMB (**a**) and OPD (**b**) substrate, respectively. The error bars represent the standard deviation of three measurements.

The effect of pH value (pH 3.0–8.0) on the OD_660nm_ for TMB and OD_450nm_ for OPD was studied, as shown in Figure 3a,b. Both the OD_660nm_ for TMB and the OD_450nm_ for OPD reached their maximum values when the pH value was 4.0. This optimum pH for the reaction of V_2_O_5_ nanozymes with TMB is consistent with the value reported in the literature [10]. Therefore, pH 4.0 was selected for H_2_O_2_ and glucose detection.

### 3.4. Effect of Buffers

**Figure 4:**
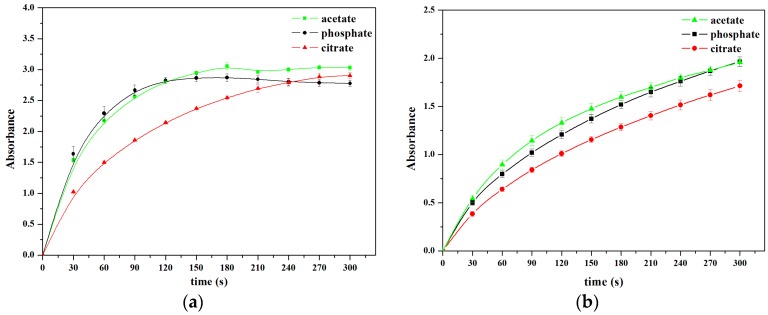
Activities of V_2_O_5_ nanozymes in, pH 4.0, 0.2 M buffers, with TMB (**a**) and OPD (**b**), respectively. The error bars represent the standard deviation of three measurements.

As shown in Figure 4, V_2_O_5_ nanozymes were incubated for as long as 300 s in pH 4.0, 0.2 M buffers, including acetate, phosphate, and citrate. The V_2_O_5_ nanozymes were more active in 0.2 M NaOAc-HOAc buffer solutions than in other buffers. Up to 300 s, the buffer order of peroxidase activity of V_2_O_5_ nanozymes is acetate > citrate > phosphate for the TMB substrate, whereas the order is acetate ≈ phosphate > citrate for the OPD substrate. Thus, the acetate buffer solution was taken as the optimal reaction solution for H_2_O_2_ and glucose determination.

### 3.5. Effect of V_2_O_5_ Nanozymes Concentrations

**Figure 5:**
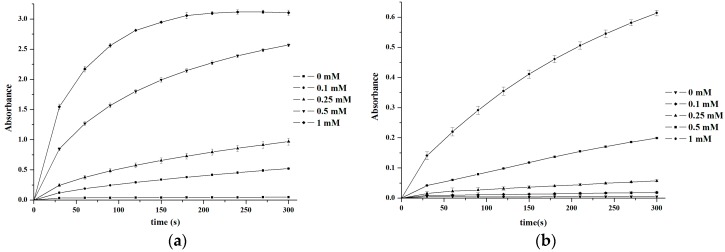
Effects of the V_2_O_5_ nanozymes concentrations ranging from 0 up to 1 mM in pH 4.0 NaOAc-HOAc buffer solution, with TMB (**a**) and OPD (**b**) respectively.

As shown in Figure 5, the OD_660nm_ and OD_450nm_ values of the system increased gradually with the concentration of V_2_O_5_ nanozymes. Then the system reached its maximum OD_660_ value when the concentration was 1 mM. Hence, 1 mM V_2_O_5_ nanozymes was chosen for the assay system.

### 3.6. Calibration Curve for H_2_O_2_ Detection

**Figure 6:**
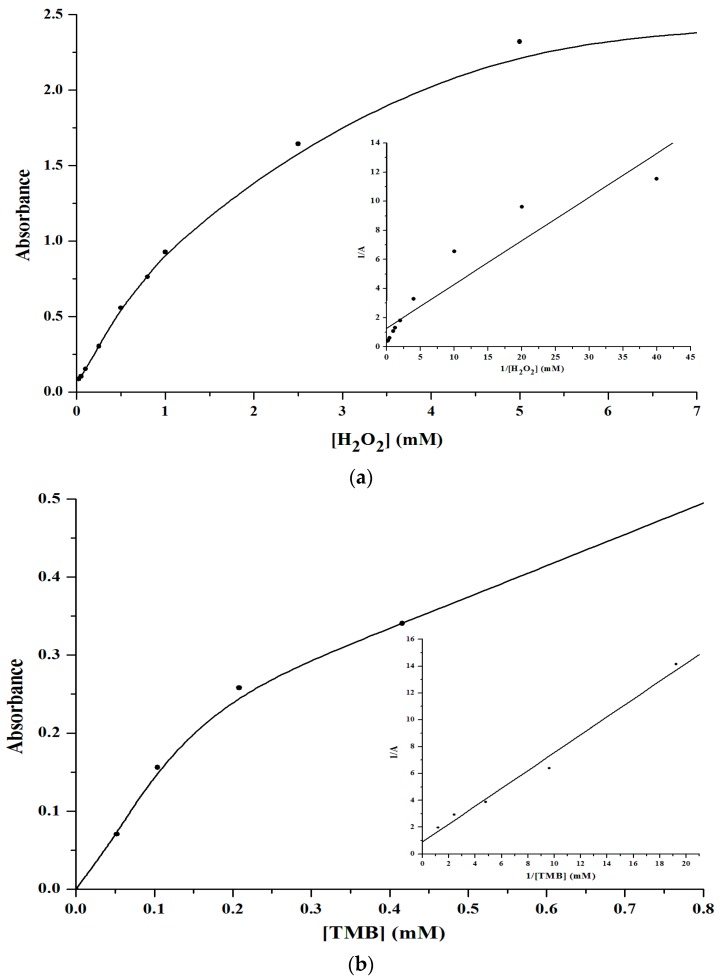
Steady-state kinetic assay and catalytic mechanism of V_2_O_5_ nanozymes as peroxidase mimic.

In a certain range of H_2_O_2_ concentrations, typical Michaelis-Menten curves (Figure 6) can be obtained for V_2_O_5_ nanozymes. *K*_M_ and *V*_max_ were obtained using a Lineweaver-Burk plot. A comparison of the kinetic parameters of V_2_O_5_ nanozymes, Fe_3_O_4_ magnetic nanoparticles (MNPs), and HRP is given in Table 1. The *K*_M_ and *V*_max_ of V_2_O_5_ nanozymes with TMB are 0.738 mM and 1.85 × 10^−5^ M·s^−1^, respectively. The *K*_M_ and *V*_max_ of V_2_O_5_ nanozymes with H_2_O_2_ are 0.232 mM and 1.29 × 10^−5^ M·s^−1^, respectively. The *K*_cat_ of V_2_O_5_ nanozymes with TMB and H_2_O_2_ are 18.46 s^−1^ and 12.91 s^−1^, respectively, and the *K*_cat_/*K*_M_ values of V_2_O_5_ nanozymes with TMB and H_2_O_2_ are 25.02 mM^−1^·s^−1^ and 55.55 mM^−1^·s^−1^, respectively. The *K*_M_ values show that V_2_O_5_ nanozymes have higher affinity for hydrogen peroxide compared with HRP, but less affinity to TMB compared with HRP. The apparent K_m_ value of V_2_O_5_ nanozymes with TMB as the substrate was higher than that for HRP, consistent with the observation that a higher TMB concentration was required to achieve the maximal activity for V_2_O_5_ nanozymes. The apparent *K*_M_ value of V_2_O_5_ nanozymes with H_2_O_2_ as the substrate was significantly lower than HRP and Fe_3_O_4_ MNPs. From the Table data, it can be concluded that the V_2_O_5_ nanozymes have a higher activity than HRP and Fe_3_O_4_ MNPs.

**Figure 7:**
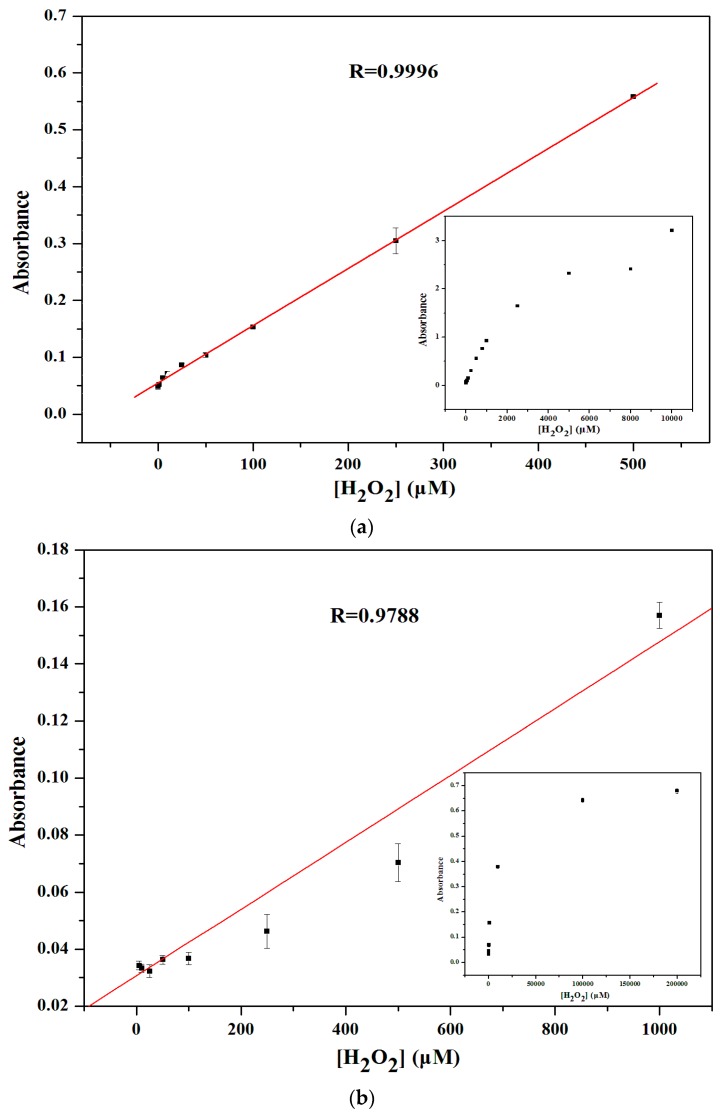
Linear calibration plot for H_2_O_2_ from 1 to 500 μM in, pH 4.0, 0.2 M NaOAc-HOAc buffers, with TMB (**a**) and OPD (**b**) respectively (*p* < 0.05). The inset shows dependence of the absorbance on the concentration of H_2_O_2_.

Under the optimized reaction conditions, calibration curves of H_2_O_2_ were plotted (Figure 7). The correlations of absorbance intensity with all H_2_O_2_ concentrations were obtained at 660 nm for TMB substrate and 450 nm for OPD substrate. There relationships between the absorbance intensities and the concentrations of H_2_O_2_ are linear over the range of 0–10 mM (TMB), and 0–0.2 M (OPD) with correlation coefficients 0.9996 (*p* < 0.05) and 0.9788 (*p* < 0.05), respectively. The results show that TMB as substrate exhibited a better response. It is supposed that the maximum absorbance peak of the oxidation of OPD is nearly equal to that of V_2_O_5_ nanozymes, so the absorbance peak of V_2_O_5_ interferes with the H_2_O_2_ sensing. The limit of detection (3σ, LOD) is 1 μM. In addition, the analytical performance of V_2_O_5_ nanozymes as peroxidase mimetics was compared with other nanozymes, as summarized in Table 2. By comparing with other nanozymes, it was revealed that the sensor has a wider linear range and higher sensitivity.

### 3.7. Calibration Curve for Glucose Detection

**Figure 8:**
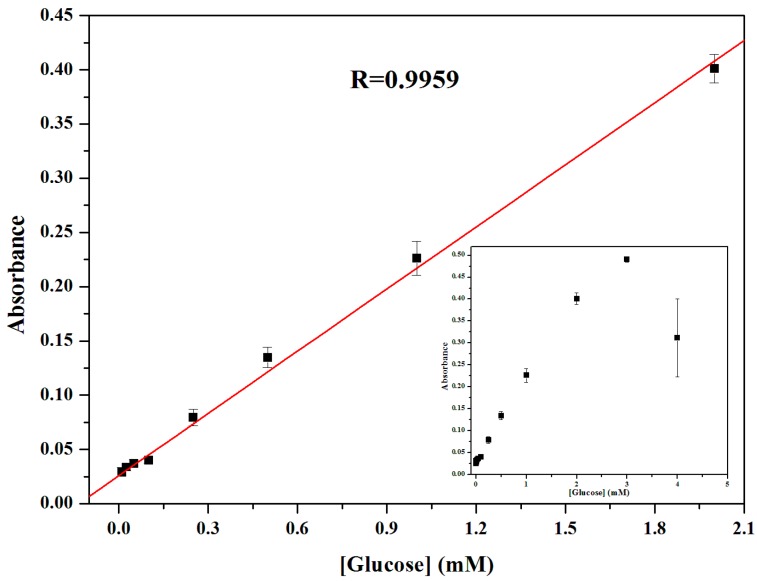
Linear calibration plot for glucose from 10 to 2000 μM (*p* < 0.05). The inset shows dependence of the absorbance on the concentration of glucose in the range from 0 to 4 mM.

It is well known that glucose oxidase (GOx) catalyzes the oxidation of glucose to gluconolactone and this property has been developed for the fabrication of glucose sensors with high sensitivity and selectivity. Due to the fact that GOx would be denatured in pH 4.0 buffer, the glucose detection was performed in two separate steps as mentioned in the Methods section. The inset of Figure 8 shows the dependence of the absorbance at 660 nm on the concentration of glucose from 0 to 4 mM. The corresponding calibration plot was shown in Figure 8. The linear detection range is from 10 to 2000 μM with the correlation coefficients, 0.9959 (*p* < 0.05). The limit of detection (3σ, LOD) is 10 μM.

Using this method, glucose was detected in patients’ blood samples. According to the linear calibration curve, the concentration of glucose in the diabetic blood sample is 15.396 mM. The general range of random plasma glucose concentration in diabetic persons is more than 11.1 mM. Therefore, this colorimetric method is applicable to real samples to determine glucose concentration.

## 4. Conclusions

In summary, a new V_2_O_5_ nanozymes-based colorimetric assay has been developed for H_2_O_2_ and glucose detection. Under the optimal reaction conditions, the method showed good responses toward H_2_O_2_ with a linear range from 1 to 500 μM. The LOD value is 1 μM. Determination of glucose was achieved from 10 to 2000 μM with a LOD of 10 μM. The result shows that the proposed assay method for H_2_O_2_ and glucose based on V_2_O_5_ nanozymes has a wide linear range, and is simple, fast, and sensitive.

## Figures and Tables

**Table 1:** Comparison of the K_m_ and V_max_ of V_2_O_5_ nanozymes, Fe_3_O_4_ MNPs, and HRP.

Nanozymes	Substrate	*K*_M_ (mM)	*V*_max_ (M·s^−1^)
V_2_O_5_ nanozymes	TMB	0.738	1.85 × 10^−5^
V_2_O_5_ nanozymes	H_2_O_2_	0.232	1.29 × 10^−5^
Fe_3_O_4_ MNPs	TMB	0.434	10.00 × 10^−8^
Fe_3_O_4_ MNPs	H_2_O_2_	154	9.78 × 10^−8^
HRP	TMB	0.434	1.24 × 10^−8^
HRP	H_2_O_2_	3.70	2.46 × 10^−8^

**Table 2:** Comparison of analytical performance for H_2_O_2_ detection of various nanozymes.

Nanozymes	Linear Range	Limit of Detection	Ref.
H_4_SiW_12_O_40_	1–20 μM	0.4 μM	[31]
H_3_PW_12_O_40_	0.134–67 μM	0.134 μM	[32]
Fe_3_O_4_ MNPs	1–100 μM	0.5 μM	[33]
Pt-DNA complexes	0.979–17.6 mM	0.392 mM	[34]
HRP	1–60 μM	1 μM	[35]
V_2_O_5_ nanozymes	1–500 μM	1 μM	This work

## Abbreviations

GOx: glucose oxidase
HRP: horseradish peroxidase
OPD: *o*-phenylenediamine
TMB: 3,3′,5,5′-tetramethylbenzidine
LOD: limit of detection
V_max_: maximum initial velocity
K_m_: Michaelis-Menten constant
